# Comparison of Simplified Creatinine Index and Systemic Inflammatory Markers for Nutritional Evaluation of Hemodialysis Patients

**DOI:** 10.3390/nu13061870

**Published:** 2021-05-30

**Authors:** Ming-Tsun Tsai, Wei-Cheng Tseng, Shuo-Ming Ou, Kuo-Hua Lee, Chih-Yu Yang, Der-Cherng Tarng

**Affiliations:** 1 Department of Medicine, Division of Nephrology, Taipei Veterans General Hospital, Taipei 11217, Taiwan; mingtsun74@gmail.com (M.-T.T.); wctseng@gmail.com (W.-C.T.); okokyytt@gmail.com (S.-M.O.); dadabim3520@gmail.com (K.-H.L.); cyyang3@vghtpe.gov.tw (C.-Y.Y.); 2 Institute of Clinical Medicine, School of Medicine, National Yang Ming Chiao Tung University, Taipei 11221, Taiwan; 3 Center for Intelligent Drug Systems and Smart Bio-Devices (IDS2B), Hsinchu 30010, Taiwan; 4 Department and Institute of Physiology, National Yang Ming Chiao Tung University, Taipei 11221, Taiwan

**Keywords:** hemodialysis, nutritional screening, protein-energy wasting, simplified creatinine index, systemic inflammation

## Abstract

Protein-energy wasting (PEW) is associated with adverse outcomes in hemodialysis patients. This study compares the simplified creatinine index (SCI) and circulating inflammatory markers as nutritional screening tools for hemodialysis patients. Maintenance hemodialysis patients (230 total patients, 34.8% women, 64.0 ± 14.3 years old) from a tertiary medical center were assessed for demographic data, body composition analysis, biochemistry tests, and circulating inflammatory biomarkers. The SCI was calculated using Canaud’s formula. Reduced fat-free mass index (FFMI), a surrogate of lean body mass, was identified according to the European Society for Clinical Nutrition and Metabolism guidelines. Nutritional status was assessed by the geriatric nutritional risk index (GNRI) and International Society of Renal Nutrition and Metabolism (ISRNM) criteria. Multivariate logistic regression revealed independent risk factors for low FFMI and malnutrition. Of the patients, 47.4% had low FFMI. Patients with a reduction in FFMI tended to be older females with lower body mass index, SCI, and GNRI scores but significantly higher levels of interleukin-6 (IL-6), tumor necrosis factor alpha (TNF-α), and IL-8. SCI was found to be an independent predictor for reduced FFMI (OR 0.57, 95% CI 0.40–0.81) and presence of PEW according to ISRNM criteria (OR 0.38, 95% CI 0.21–0.68). Although a positive association between systemic inflammatory markers and low FFMI was observed, this association disappeared in multivariate analysis. Moreover, the inflammatory markers examined in this study were not associated with malnutrition after adjusting for potential confounders. Compared with markers of systemic inflammation, SCI achieved better performance in assessing the nutritional status of hemodialysis patients.

## 1. Introduction

Protein-energy wasting (PEW) is a common complication associated with adverse clinical events in patients with advanced chronic kidney disease (CKD) [1]. Individuals with kidney dysfunction commonly suffer from inadequate nutrient intake, chronic inflammation, comorbidities, metabolic derangements, accumulation of uremic toxins, and protein loss during dialysis [2,3], which can cause PEW, accelerate the loss of lean body mass, and increase the risk of subsequent disability and mortality [4,5]. Therefore, accurate measurement of skeletal muscle mass, the main component of lean mass, is widely implemented to assess the nutritional health of patients with CKD.

Several instruments, including magnetic resonance imaging, computed tomography, dual-energy X-ray absorptiometry, and bioelectrical impedance analysis (BIA), have been developed to estimate skeletal muscle mass. However, there are disadvantages to each of these methods, including high cost, accessibility, non-portability, and interference caused by abnormal hydration status [6,7,8]. By contrast, the creatinine kinetic modeling (CKM)-derived creatinine index has been validated as a convenient and reliable tool for assessing lean body mass and nutritional status in patients receiving dialysis [9,10]. CKM is based on the principle that the body generates creatinine in direct proportion to muscle mass in stable dialysis patients who consume protein regularly. Despite its simplicity, several challenges occur when using the kinetically derived creatinine index. Computing the creatinine generation rate requires collecting dialysate samples. The traditional creatinine index formula is too complicated for application in daily clinical practice [11,12].

Recently, Canaud et al. developed a simplified creatinine index (SCI) equation for estimating the skeletal muscle mass of hemodialysis patients [13]. The SCI takes into account patient demographic information and dialysis dosage and distinguishes lean body mass as a predictors of mortality [14,15]. Researchers found that the long-term predictive value of SCI was comparable to that of muscle function, including gait speed and hand-grip strength [16]. Furthermore, the trajectory of lean body mass assessed using SCI is an independent risk factor for mortality and provides additional prognostic information to the changes in body mass index (BMI) in patients undergoing maintenance hemodialysis [17,18]. These findings suggest that SCI is a simple and useful tool for early identification of muscle wasting.

CKD is characterized by persistent, low-grade inflammation, which stimulates muscle protein degradation while suppressing appetite, inducing insulin resistance, and increasing resting energy expenditure [19,20]. In patients on maintenance dialysis, elevated levels of several circulating inflammatory cytokines are associated with increased risk of PEW and its related complications, such as cardiovascular diseases (CVDs), infectious diseases, and falls [21,22]. Moreover, activation of ubiquitin-proteasome system (UPS) is a major cause of muscle wasting in patients with CKD [23]. There was abundant evidence that certain inflammatory mediators, such as tumor necrosis factor alpha (TNF-α) and interleukin-6 (IL-6), were involved in the activation of UPS [24]. Despite the above-mentioned findings, there have been few studies examining the validity and reliability of using inflammatory markers to assess nutritional status in patients with CKD stages 3–5, including those receiving maintenance dialysis [25] (pp. 31–33). These studies were mainly cross-sectional and examined only the correlations between levels of inflammatory mediators and nutritional indices. As stated by the latest KDOQI recommended guidelines for nutrition, further studies are needed to address the role of systemic inflammatory markers in the diagnosis and management of malnutrition in this patient population [25]. Therefore, the aim of this study was to compare the performance of SCI and circulating inflammatory factors to assess lean body mass and the nutritional status of hemodialysis patients. A set of plasma inflammatory biomarkers, such as TNF-α, IL-6, IL-8, monocyte chemoattractant protein-1 (MCP-1/CCL2), and soluble Toll-like receptor 4 (sTLR4), were used to assess systemic inflammation. We investigated whether SCI more accurately detects lower lean tissue mass and malnutrition than do inflammatory mediators in a cohort of hemodialysis patients.

## 2. Materials and Methods

### 2.1. Study Design and Participants

This was a cross-sectional study conducted in Taipei Veterans General Hospital, Taiwan. Study participants were recruited from 1 January to 31 December 2020. Initially, 251 patients aged >20 years who had been on maintenance hemodialysis three times per week for ≥3 months were recruited. All patients had lost residual renal function (the residual renal urea clearance <2 mL/min). Exclusion criteria were as follows: dialysis treatment time <12 h/week (*n* = 5), patients with a prior amputation (*n* = 2), patients with a pacemaker or implantable cardioverter-defibrillator (*n* = 2), and malignancy (*n* = 3), infectious disease, sepsis (*n* = 5), or hepatobiliary disease (*n* = 4). Finally, 230 clinically stable patients were included in this study. The study protocol was approved by the institutional review board of Taipei Veterans General Hospital (protocol code 2019-07-026BC and date of approval: 5 July 2019). Written informed consent was obtained from all participants, and all aspects of the study complied with the Declaration of Helsinki.

A thorough medical history was obtained for each patient. CVD was defined as a medical history and clinical finding of coronary artery disease, stroke, and/or peripheral vascular disease. Diabetes mellitus was defined as past or current users of oral hypoglycemic agents and/or insulin. Hypertension was defined as a blood pressure of greater than 140/90 mmHg or patient use of antihypertensive medication.

### 2.2. Laboratory Measurements

Blood samples were collected from patients who had fasted overnight prior to starting dialysis, and plasma was separated and stored at −80 °C until analysis. Plasma levels of IL-6, TNF-α, IL-8, and MCP-1 were measured using the Bio-Plex Multiplex Immunoassay System (Bio-Rad) based on a previously published protocol [26]. Human sTLR4 levels were detected using an enzyme-linked immunoassay kit from Elabscience (Wuhan, China) following the manufacturer’s instructions. Each sample was tested in duplicate to verify results. Serum levels of albumin, calcium, phosphate, total cholesterol, urea, and creatinine were determined with a Hitachi 7600 autoanalyzer (Hitachi, Tokyo, Japan). Adequacy of dialysis was estimated by single-pool Kt/V for urea (spKt/V_urea_) using the Daugirdas equation [27]. Pre-dialysis blood pressure was measured from the nonaccess arm after the patient had rested 5 min in a seated position before needle insertion for hemodialysis. The mean arterial pressure was calculated by adding one-third of the pulse pressure to diastolic pressure.

### 2.3. Calculation of SCI

Canaud’s formula for calculating SCI was developed based on age, sex, pre-dialysis serum creatinine concentration, and spKt/V_urea_ [13]:

SCI (mg/kg/day) = 16.21 + 1.12 × (1 if male; 0 if female) − 0.06 × age (years) − 0.08 × spKt/V_urea_ + 0.009 × pre-dialysis serum creatinine concentration (μmol/L) (1)

### 2.4. Evaluation of Lean Body Mass

Body composition was determined using multifrequency bioimpedance analysis (MFBIA) (InBody S10 machine; InBody, Seoul, Korea). The InBody S10 provides impedance measurements at 6 different frequencies (1, 5, 50, 250, 500, and 1000 kHz) for each segment of the body (trunk, left arm, right arm, left leg, and right leg) [28]. Patients underwent MFBIA examination at approximately 30 min post-dialysis on the day of blood sampling. Fat mass, fat-free mass, and extracellular water/total body water (ECW/TBW) were measured through equations and algorithms developed by the manufacturer and normalized for height. Technically, lean body mass differs from fat-free mass because fat in the bone marrow and other internal organs are included in lean body mass; however, this accounts for a tiny fraction of body weight [29]. In this study, fat-free mass index (FFMI, kg/m^2^) was therefore used as a surrogate marker for estimating lean body mass, and reduced lean body mass was defined as a FFMI <17 kg/m^2^ in men and <15 kg/m^2^ in women, according to the European Society of Clinical Nutrition and Metabolism (ESPEN) definition [30].

### 2.5. Assessment of Nutritional Status

The nutritional statuses of the study participants were assessed using the geriatric nutritional risk index (GNRI) and PEW criteria as proposed by the International Society of Renal Nutrition and Metabolism (ISRNM) [31,32]. The GNRI was obtained using the following formula:

GNRI = [14.89 × serum albumin (g/dl)] + [41.7 × (dry weight/ideal body weight)](2)

Ideal body weight was calculated from the height and ideal BMI of 22. The cutoff value of GNRI indicating malnutrition was 98, according to previous literatures [31,33,34].

The ISRNM expert panel established four main categories for diagnosing PEW: serum biochemistry, body mass, muscle mass, and dietary intake. Normalized protein catabolic rate (nPCR) is used as a surrogate for daily dietary protein intake and calculated as previously described in detail [27]. The diagnostic criteria for PEW in this study were as follows: (1) biochemical parameter: serum albumin <3.8 g/dL; (2) body mass: BMI < 23 kg/m^2^; (3) muscle mass: muscle wasting is defined as a decrease of more than 5% in the BIA-determined muscle mass, measured on ≥2 occasions, at least 3 months apart; and (4) dietary intake: nPCR < 0.8 g/kg/day. At least 3 of these criteria must be met for a PEW diagnosis.

### 2.6. Statistical Analysis

The sample size needed for estimating the prevalence of PEW in this study was calculated according to the following formula [35]:

*n* = Z_1−α/2_^2^*p*(1 − *p*)/d^2^ (3)

where *n* is the sample size, Z_1−α/2_ is standard normal variate (a *P* value less than 0.05 is considered significant; hence, 1.96 is used in this formula), *p* is expected prevalence (a recent meta-analysis demonstrated the median prevalence of PEW was 43% in maintenance hemodialysis patients [36]), and d is precision (the precision in this study is 0.1). Therefore, we had to take at least 94 subjects for this cross-sectional study.

Clinical variables were expressed as the frequency and percentage for categorical data and the mean ± SD or median and interquartile range for continuous data with or without normal distribution, respectively. The study population was divided into two subgroups depending on the amount of lean body mass. Comparisons of the groups were conducted using chi-square test, Student’s *t* test, or Mann–Whitney U test. The association between variables was determined using Spearman’s correlation analysis. Multivariate logistic regression was used to reveal independent risk factors for low lean body mass and malnutrition; the independent parameters listed in Table 1 that had *P* values < 0.25 in univariate analysis and those considered to be clinically important were covariates. Data were stratified by gender to clarify factors affecting FFMI in both sexes. Receiver operating characteristic (ROC) curves were constructed, and the area under the ROC curve (AUC) was calculated to assess the capability of SCI to detect decreases in lean body mass. *P* values < 0.05 were statistically significant. Data were analyzed using SPSS v23.0 software (SPSS Inc., Chicago, IL, USA).

## 3. Results

### 3.1. Baseline Characteristics of Study Population

The 230 enrolled participants had a mean age of 64.0 ± 14.3 years, and 34.8% were women. Lean body mass, estimated by FFMI in this study, is known to be a useful marker for assessing nutritional status among hemodialysis patients [17]. Therefore, to assess the relationship between lean mass and clinical features, the study participants were divided into two groups according to the presence or absence of low lean body mass. Based on the ESPEN cutoff values, 109 subjects (47.4%) had low lean body mass. Table 1 summarizes the clinical characteristics, body composition parameters, and laboratory findings of groups with normal and reduced FFMI. Patients with low FFMI tended to be older females with a longer dialysis vintage, higher spKt/V_urea_, and lower mean arterial pressure. Compared with subjects with normal FFMI, the low FFMI group was more likely to have a higher ECW/TBW ratio and lower BMI, SCI, and GNRI scores. A higher percentage of patients with PEW had decreased lean body mass. Furthermore, women with low lean body mass had significantly lower FMI than women with normal lean body mass. Laboratory results revealed that patients with reduced lean body mass had significantly lower levels of serum albumin and phosphorus but significantly higher levels of IL-6, TNF-α, and IL-8.

### 3.2. Correlation Analysis between Body Composition, Circulating Inflammatory Markers, GNRI, and SCI in Hemodialysis Patients

Spearman’s correlation was performed to determine the relationship among SCI, circulating inflammatory markers, and various nutritional parameters. Correlation analysis revealed that SCI was positively correlated with BMI, FFMI, and GNRI and negatively correlated with IL-6, TNF-α, IL-8, MCP-1, and sTLR4, as presented in Table 2. All inflammatory markers were significantly correlated (all ρ  >  0.2, *P* < 0.001) and showed similar tendencies regarding correlations with FFMI and GNRI. There was no correlation between proinflammatory mediators and BMI. Moreover, FMI, an estimate of adiposity, was not associated with the SCI or plasma levels of inflammatory markers.

### 3.3. Factors Associated with Reduced Lean Body Mass among Hemodialysis Patients

Based on univariate logistic regression analysis, age, dialysis vintage, ECW/TBW, BMI, mean arterial pressure, GNRI, serum calcium and phosphorus levels, SCI, and IL-8 were associated with low lean body mass (Table 3). After multivariate adjustment, BMI and SCI remained the only independent predictors of reduced lean body mass among hemodialysis patients.

**Figure 1:**
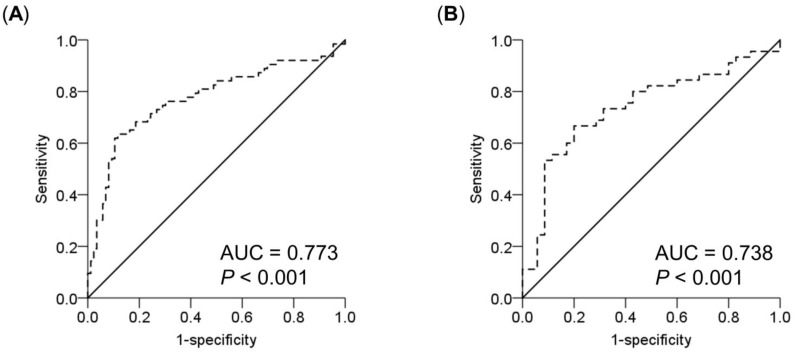
Receiver operating characteristic (ROC) curves of simplified creatinine index for prediction of reduced lean body mass in male (**A**) and female (**B**) hemodialysis patients. The area under the curve (AUC) represents the probability to discriminate a reduction in lean body mass in these subjects.

Because there were gender differences in lean body mass, we performed a separate analysis by gender (Table 4). Consistent with the above-mentioned findings, the SCI remained significantly associated with a reduction in FFMI in either sex after adjusting for other factors. ROC analysis was used to evaluate the ability of SCI to identify and assess low FFMI (Figure 1). The results showed that the AUCs for SCI to discriminate normal and reduced lean body mass were 0.773 (95% CI, 0.692–0.855; *P* < 0.001) and 0.738 (95% CI, 0.627–0.850; *P* < 0.001) for men and women, respectively. To detect a reduction in lean body mass, the optimal SCI cutoff values of ≤20.37 and ≤18.39 mg/kg/day for men and women yielded sensitivities of 63.5% and 66.7% and specificities of 88.4% and 80.0%, respectively.

### 3.4. Factors Associated with PEW among Hemodialysis Patients

Nutritional status was assessed based on PEW criteria, and multivariate logistic regression analyses were performed to determine predictive variables for PEW status (Table 5). While adjusting for potential confounders, we found that SCI remained an independent predictor for the presence of PEW according to ISRNM criteria. In contrast, among the inflammatory markers examined in this study, none of them were associated with PEW in the multivariate analysis.

## 4. Discussion

Our study indicates that PEW was highly prevalent among patients with end-stage renal disease (ESRD). The present study provides evidence that the SCI provides a simple and efficient method for assessing lean body mass and nutritional status in hemodialysis patients, which supports previous findings. Moreover, we did not find an association between the nutritional status and the indices of inflammation. Therefore, this study showed that SCI has a better performance for identifying hemodialysis patients at risk for malnutrition.

Muscle atrophy influences energy metabolism, locomotion, breathing, and swallowing, which contributes to the prognosis of CKD patients [37]. Therefore, assessment of skeletal muscle mass is indicated for CKD patients who are at risk for frailty and PEW. However, there is no consensus on which method is best for measuring and defining decreased muscle mass, especially clinically [38]. SCI can be readily calculated from variables that are measured regularly in routine hemodialysis. Numerous studies and our own observations have shown that amount of lean body mass derived from SCI and the bioimpedance technique were significantly correlated, although the former may overestimate lean body mass compared with the measured method [18,39]. A lower SCI was associated with an increased risk for fractures, cardiovascular events, and mortality [15,18,39]. Moreover, it was noted that accelerated decline of SCI in hemodialysis patients may indicate poorer prognosis [17,18]. Hence, results from these studies demonstrate that SCI is a valid tool for assessing nutritional status in the hemodialysis patients not only in epidemiological surveys but in clinical practice and research.

Our results demonstrated significant increases in IL-6, TNF-α, and IL-8 in the plasma of hemodialysis patients with a reduction in FFMI compared with those with normal FFMI; however, markers of systemic inflammation are less reliable than SCI for the assessment of lean body mass and nutritional status. There is significant evidence showing that the inflammatory markers described above are involved in chronic stress-induced muscle wasting by activating transcriptional networks that promote the expression of atrophy-related genes (atrogenes) [40,41]. For example, TNF-α and IL-6 can induce muscle atrophy by inducing myostatin through the JAK/STAT3 pathway [42]. In addition to JAK/STAT, TNF-α activates p38 MAPK and NF-κB signaling pathways to increase the expression of MAFbx/atrogin-1 and MuRF1, which are two major classes of muscle-specific E3 ubiquitin ligases [43]. Despite this, the association between circulating inflammatory markers and the development of muscle wasting in CKD patients was not consistent [44,45].

Although circulating inflammatory biomarkers were associated with an increased risk of sarcopenia and PEW in previous studies, they may not adequately reflect disease severity in skeletal muscles at the individual patient level. Skeletal muscle can produce different types of myokines that have endocrine and paracrine actions to exert multiple beneficial effects on human health [46]. Muscle wasting in patients with chronic inflammation may lead to insufficient myokine signaling, which alters immune cell function and perpetuates pre-existing inflammatory responses [47]. Therefore, distinct processes and consequences of systemic versus local inflammation in muscle cells may explain inconsistencies between studies. The updated KDOQI nutrition guideline states that systemic inflammatory markers may provide information about underlying causes of PEW in adults with CKD, although their usefulness for nutrition assessment has not been determined [25]. Recently, the development of high-throughput proteomic platforms for large-scale analyses of common neuromuscular disorders has identified numerous candidate proteins for improving diagnostic and prognostic accuracy in the field of muscle pathology [48]. However, the relationships among specific biomarkers of muscle metabolism, inflammation, regeneration, and subsequent adverse events in patients undergoing hemodialysis are still unclear and require further elucidation in large clinical trials.

This study has some limitations. First, the number of participants was small, so further studies using a larger population are needed to validate these findings. Second, the cross-sectional design of the study makes it difficult to establish a causal relationship between variables. Therefore, the associations we found should be interpreted with caution. Third, residual confounding may still exist despite adjustments for potential confounding factors. Fourth, we did not detect levels of C-reactive protein (CRP), a more clinically accessible inflammatory marker, in our study participants. As a result, this study did not exclude the utility of CRP for screening and evaluating the nutritional status in hemodialysis patients. Fifth, BIA-derived FFMI may be influenced by volume status. Hence these findings should be interpreted with caution in edematous patients. Finally, GNRI is a simple and accurate method for predicting long-term outcomes of chronic hemodialysis patients. However, it may provide less comprehensive information compared to other nutritional screening indices, such as the malnutrition-inflammation score (MIS) [49]. The performance of SCI needs to be compared to that of MIS in future studies. Integration of all data available from the full nutrition assessment would allow for early identification and timely treatment of patients who are at risk of malnutrition.

## 5. Conclusions

In conclusion, PEW and loss of lean body mass are common complications of ESRD. Compared with markers of systemic inflammation, SCI achieves better performance in assessing the nutritional statuses of patients undergoing hemodialysis.

## Figures and Tables

**Table 1:** Baseline characteristics of hemodialysis patients with and without decreased lean body mass.

Variables ^a^	Fat-Free Mass Index (FFMI, kg/m^2^)		
	Normal (Men: ≥17; Women: ≥15)	Low (Men: <17; Women: <15)	*P* Value
Number of patients	*n* = 121	*n* = 109	
Demographic and clinical characteristics			
Age, years	59.9 ± 11.5	68.6 ± 15.7	<0.001
Women, *n* (%)	35 (28.9)	45 (41.3)	0.049
Body height, cm	165.0 (158.7–169.8)	161.0 (154.2–166.5)	<0.001
Body weight, kg	68.8 (63.4–78.9)	57.2 (50.1–65.3)	<0.001
Dialysis vintage, months	45.0 (21.5–63.0)	54.0 (36.5–128.0)	0.006
spKt/V_urea_	1.42 ± 0.21	1.65 ± 0.25	<0.001
nPCR, g/kg/day	1.15 ± 0.22	1.14 ± 0.27	0.763
Hypertension, *n* (%)	109 (90.1)	94 (86.2)	0.366
Diabetes mellitus, *n* (%)	69 (57.0)	60 (55.0)	0.763
CVD ^b^, *n* (%)	55 (45.5)	54 (49.5)	0.535
Mean arterial pressure, mmHg	105 ± 17	98 ± 18	0.001
Body composition and nutritional scores			
BMI, kg/m^2^	26.1 (23.3–28.9)	22.6 (20.2–25.0)	<0.001
Men	25.3 (23.2–29.2)	23.0 (20.3–25.7)	<0.001
Women	27.0 (24.1–28.8)	22.4 (19.7–24.5)	<0.001
FFMI, kg/m^2^	17.8 (17.0–19.2)	14.7 (13.9–16.0)	<0.001
Men	18.6 (17.7–19.6)	15.8 (14.8–16.5)	<0.001
Women	16.0 (15.4–16.5)	14.1 (13.3–14.5)	<0.001
FMI, kg/m^2^	8.2 (5.3–11.0)	8.0 (5.1–10.0)	0.426
Men	6.9 (4.7–10.2)	7.8 (4.9–9.8)	0.796
Women	10.5 (7.8–12.8)	8.1 (6.0–10.5)	0.006
ECW/TBW, %	39.9 (38.9–41.0)	40.4 (39.5–41.5)	0.005
SCI, mg/kg/day	21.55 (19.94–23.03)	19.08 (17.13–20.65)	<0.001
Men	22.36 (21.07–23.47)	19.88 (18.76–21.38)	<0.001
Women	19.48 (18.62–20.44)	17.26 (16.46–19.16)	<0.001
GNRI	102.7 (99.7–106.5)	99.8 (95.4–103.0)	<0.001
GNRI ≤ 98, *n* (%)	19 (15.7)	40 (36.7)	<0.001
Presence of PEW ^c^, *n* (%)	15 (12.4)	30 (27.5)	0.003
Laboratory parameters			
Albumin, g/dL	4.1 (3.9–4.4)	4.0 (3.8–4.3)	0.042
Calcium, mg/dL	9.2 (8.7–9.8)	9.1 (8.4–9.6)	0.058
Phosphorous, mg/dL	5.3 (4.5–6.3)	4.8 (3.9–5.6)	<0.001
Total cholesterol, mg/dL	142.5 (124.0–168.8)	138.5 (122.0–162.0)	0.305
Glucose, mg/dL	118.5 (95.3–157.5)	130.0 (104.0–179.8)	0.164
Hemoglobin, g/dL	10.0 ± 1.4	9.9 ± 1.2	0.895
Inflammatory markers			
IL-6, pg/mL	10.62 (5.58–18.87)	14.86 (8.80–24.23)	0.004
TNF-α, pg/mL	2.34 (1.03–4.19)	3.24 (1.32–5.71)	0.012
IL-8, pg/mL	4.04 (1.03–19.12)	15.32 (1.68–36.51)	0.003
MCP-1, pg/mL	164.03 (127.41–223.92)	183.99 (147.46–252.23)	0.065
sTLR4, pg/mL	156.28 (115.63–253.55)	182.41 (126.25–293.93)	0.097

**Abbreviations:** BMI, body mass index; CVD, cardiovascular disease; ECW/TBW, extracellular water/total body water ratio; FFMI, fat-free mass index; FMI, fat mass index; GNRI, geriatric nutritional risk index; IL-6, interleukin-6; IL-8, interleukin-8; MCP-1, monocyte chemoattractant protein-1; nPCR, normalized protein catabolic rate; PEW, protein-energy wasting; SCI, simplified creatinine index; spKt/V_urea_, single pool Kt/V_urea_; sTLR4, soluble Toll-like receptor 4; TNF-α, tumor necrosis factor-α. ^a^ Variables are expressed as *n* (%) for categorical data and as mean values ± SD or medians and interquartile ranges for continuous data with or without a normal distribution, respectively. ^b^ History of CVD consisted of coronary artery disease, cerebrovascular disease, and peripheral arterial disease. ^c^ Protein-energy wasting was diagnosed according to the International Society of Renal Nutrition and Metabolism (ISRNM) expert panel.

**Table 2:** Correlation among body composition, circulating inflammatory markers, geriatric nutritional risk index, and simplified creatinine index in adults undergoing maintenance hemodialysis.

	Correlations ^a^									
*N* = 230	BMI	FFMI	FMI	GNRI	SCI	IL-6	TNF-α	IL-8	MCP-1	sTLR4
BMI	1									
FFMI	0.479 ^d^	1								
FMI	0.835 ^d^	−0.041	1							
GNRI	0.377 ^d^	0.307 ^d^	0.253 ^d^	1						
SCI	0.266 ^d^	0.645 ^d^	−0.078	0.443 ^d^	1					
IL-6	−0.062	−0.107	−0.013	−0.426 ^d^	−0.316 ^d^	1				
TNF-α	−0.039	−0.178 ^c^	0.074	−0.266 ^d^	−0.275 ^d^	0.415 ^d^	1			
IL-8	−0.133	−0.161 ^b^	−0.049	−0.331 ^d^	−0.359 ^d^	0.452 ^d^	0.631 ^d^	1		
MCP-1	0.006	−0.155 ^b^	0.101	−0.115	−0.184 ^c^	0.301 ^d^	0.378 ^d^	0.344 ^d^	1	
sTLR4	0.005	−0.121	0.061	−0.108	−0.150 ^b^	0.311 ^d^	0.356 ^d^	0.326 ^d^	0.248 ^d^	1

**Abbreviations:** BMI, body mass index; FFMI, fat-free mass index; FMI, fat mass index; GNRI, geriatric nutritional risk index; IL-6, interleukin-6; IL-8, interleukin-8; MCP-1, monocyte chemoattractant protein-1; SCI, simplified creatinine index; sTLR4, soluble Toll-like receptor 4; TNF-α, tumor necrosis factor-α. ^a^ Spearman’s correlation coefficients are shown. ^b^ *P* < 0.05. ^c^ *P* < 0.01. ^d^ *P* < 0.001.

**Table 3:** Independent predictors for reduced lean body mass among hemodialysis patients.

	Reduced Lean Body Mass			
	Univariate		Multivariate ^a^	
Variables	Odds Ratio (95% CI)	*P* Value	Odds Ratio (95% CI)	*P* Value
Age (per year)	1.05 (1.03–1.07)	<0.001	0.99 (0.95–1.03)	0.495
Gender (male:female)	0.58 (0.34–1.00)	0.050	2.92 (0.93–9.17)	0.067
Dialysis vintage (per year)	1.08 (1.02–1.15)	0.007	1.06 (0.97–1.16)	0.182
ECW/TBW (per 1%)	1.30 (1.09–1.55)	0.004	0.74 (0.53–1.05)	0.093
BMI (per 1 unit)	0.76 (0.70–0.83)	<0.001	0.73 (0.65–0.83)	<0.001
Mean arterial pressure (per 10 mmHg)	0.78 (0.66–0.91)	0.002	0.92 (0.73–1.16)	0.473
GNRI (per 1 unit increase)	0.90 (0.86–0.95)	<0.001	1.03 (0.96–1.10)	0.424
Calcium (mg/dL)	0.70 (0.51–0.96)	0.028	0.83 (0.53–1.30)	0.410
Phosphorous (mg/dL)	0.70 (0.57–0.85)	<0.001	0.98 (0.75–1.28)	0.892
Glucose (mg/dL)	1.00 (1.00–1.01)	0.170	1.01 (1.00–1.01)	0.070
SCI (per mg/kg/day)	0.68 (0.60–0.77)	<0.001	0.57 (0.40–0.81)	0.002
IL-6 levels (per pg/mL)	1.00 (1.00–1.01)	0.388	−	−
TNF-α levels (per pg/mL)	1.00 (0.99–1.01)	0.553	−	−
IL-8 levels (per pg/mL)	1.01 (1.00–1.03)	0.012	1.00 (0.99–1.01)	0.801
MCP-1 levels (per 10 pg/mL)	1.02 (1.00–1.04)	0.085	1.04 (1.00–1.07)	0.051
sTLR4 levels (per 10 pg/mL)	1.01 (1.00–1.02)	0.112	1.01 (0.99–1.03)	0.343

**Abbreviations:** BMI, body mass index; CI, confidence interval; ECW/TBW, extracellular water/total body water; GNRI, geriatric nutritional risk index; IL-6, interleukin-6; IL-8, interleukin-8; MCP-1, monocyte chemoattractant protein-1; SCI, simplified creatinine index; sTLR4, soluble Toll-like receptor 4; TNF-α, tumor necrosis factor-α. ^a^ The multivariable model is adjusted for model including age, gender, dialysis vintage, ECW/TBW, BMI, mean arterial pressure, GNRI, calcium, phosphorous, glucose, SCI, and levels of IL-8, MCP-1, and sTLR4.

**Table 4:** Multivariate logistic regression analysis of factors associated with reduced lean body mass in male and female hemodialysis patients ^a^.

	Reduced Lean Body Mass			
	Men		Women	
Variables	Odds Ratio (95% CI)	*P* Value	Odds Ratio (95% CI)	*P* Value
Age (per year)	1.01 (0.96−1.06)	0.642	0.89 (0.79−0.99)	0.038
Dialysis vintage (per year)	1.08 (0.96−1.23)	0.206	0.98 (0.81−1.18)	0.821
ECW/TBW (per 1%)	0.76 (0.50−1.15)	0.191	0.65 (0.30−1.44)	0.293
BMI (per 1 unit)	0.77 (0.67−0.89)	<0.001	0.51 (0.33−0.78)	0.002
Mean arterial pressure (per 10 mmHg)	0.96 (0.71−1.29)	0.763	1.12 (0.67−1.89)	0.662
GNRI (per 1 unit increase)	1.01 (0.92−1.11)	0.814	1.24 (1.00−1.55)	0.055
Calcium (mg/dL)	0.82 (0.45−1.50)	0.521	1.04 (0.44−2.47)	0.934
Phosphorous (mg/dL)	1.19 (0.86−1.64)	0.285	0.46 (0.22−0.95)	0.036
Glucose (mg/dL)	1.01 (1.00−1.01)	0.152	1.01 (1.00−1.02)	0.244
SCI (per mg/kg/day)	0.65 (0.45−0.95)	0.027	0.23 (0.07−0.77)	0.017
IL-8 levels (per pg/mL)	1.00 (0.99−1.01)	0.793	1.01 (0.98−1.04)	0.551
MCP-1 levels (per 10 pg/mL)	1.03 (0.99−1.08)	0.149	1.01 (0.94−1.09)	0.763
sTLR4 levels (per 10 pg/mL)	1.01 (0.99−1.03)	0.363	1.00 (0.93−1.07)	0.994

**Abbreviations:** BMI, body mass index; CI, confidence interval; ECW/TBW, extracellular water/total body water; GNRI, geriatric nutritional risk index; IL-6, interleukin-6; IL-8, interleukin-8; MCP-1, monocyte chemoattractant protein-1; SCI, simplified creatinine index; sTLR4, soluble Toll-like receptor 4; TNF-α, tumor necrosis factor-α. ^a^ The multivariable model is adjusted for model including age, gender, dialysis vintage, ECW/TBW, BMI, mean arterial pressure, GNRI, calcium, phosphorous, glucose, SCI, and levels of IL-8, MCP-1, and sTLR4.

**Table 5:** Logistic regression analyses of potential factors associated with PEW status according to ISRNM criteria in patients undergoing hemodialysis.

	Univariate		Multivariate ^a^	
Variables	Odds Ratio (95% CI)	*P* Value	Odds Ratio (95% CI)	*P* Value
Age (per year)	1.04 (1.01–1.06)	0.008	0.91 (0.85–0.97)	0.003
Gender (male:female)	0.61 (0.31–1.18)	0.138	14.99 (1.56–143.67)	0.019
nPCR (per 0.1 g/kg/day)	0.70 (0.59–0.82)	<0.001	0.63 (0.45–0.88)	0.006
BMI (per 1 kg/m^2^)	0.81 (0.73–0.89)	<0.001	0.93 (0.80–1.09)	0.367
ECW/TBW (per 1%)	1.06 (0.97–1.16)	<0.001	0.76 (0.47–1.23)	0.264
Calcium (mg/dL)	0.58 (0.39–0.86)	0.007	1.53 (0.76–3.08)	0.232
Phosphorous (mg/dL)	0.58 (0.44–0.75)	<0.001	1.25 (0.75–2.08)	0.401
Total cholesterol (mg/dL)	0.98 (0.96–0.99)	<0.001	0.98 (0.96–1.00)	0.114
Hemoglobin (g/dL)	0.64 (0.47–0.85)	0.002	0.88 (0.55–1.41)	0.598
GNRI (per 1 unit)	0.68 (0.61–0.77)	<0.001	0.69 (0.57–0.82)	<0.001
SCI (per mg/kg/day)	0.58 (0.49–0.70)	<0.001	0.38 (0.21–0.68)	0.001
IL-6 levels (per pg/mL)	1.00 (1.00–1.01)	0.213	1.00 (0.99–1.01)	0.740
TNF-α levels (per pg/mL)	1.02 (0.97–1.08)	0.392	−	−
IL-8 levels (per pg/mL)	1.01 (1.00–1.01)	0.117	1.00 (0.99−1.01)	0.400
MCP-1 levels (per 10 pg/mL)	1.02 (1.00–1.04)	0.060	1.01 (0.97−1.06)	0.674
sTLR4 levels (per 10 pg/mL)	1.01 (0.99–1.02)	0.549	−	−

**Abbreviations:** BMI, body mass index; CI, confidence interval; ECW/TBW, extracellular water/total body water; GNRI, geriatric nutritional risk index; IL-6, interleukin-6; IL-8, interleukin-8; ISRNM, The International Society of Renal Nutrition and Metabolism; MCP-1, monocyte chemoattractant protein-1; nPCR, normalized protein catabolic rate; PEW, protein-energy wasting; SCI, simplified creatinine index; sTLR4, soluble Toll-like receptor 4; TNF-α, tumor necrosis factor-α. ^a^ The multivariable model is adjusted for model including age, gender, nPCR, BMI, ECW/TBW, calcium, phosphorous, total cholesterol, hemoglobin, GNRI, SCI, and levels of IL-6, IL-8, and MCP-1.

## Data Availability

Restrictions apply to the availability of these data. Interested groups should contact Prof. Der-Cherng Tarng at dctarng@vghtpe.gov.tw to discuss access permission.
